# Development of WHO Recommendations for the Final Phase of Elimination and Prevention of Re-Establishment of Malaria

**DOI:** 10.4269/ajtmh.22-0768

**Published:** 2023-12-20

**Authors:** Kevin Marsh, Elie Akl, Jane Achan, Mohammed Alzahrani, J. Kevin Baird, Teun Bousema, Dionicia Gamboa, Marcus Lacerda, Kamini Mendis, Melissa Penny, Allan Schapira, Siv Sovannaroth, Chansuda Wongsrichanalai, Amanda Tiffany, Xiaohong Li, Erin Shutes, David Schellenberg, Pedro Alonso, The WHO Malaria Elimination Steering Group, Kim A. Lindblade

**Affiliations:** ^1^ Centre for Tropical Medicine and Global Health, University of Oxford, United Kingdom;; ^2^ Department of Internal Medicine, American University of Beirut, Lebanon;; ^3^ Department of Health Research Methods, Evidence and Impact, McMaster University, Hamilton, Canada;; ^4^ Malaria Consortium, London, United Kingdom;; ^5^ Public Health Agency, Ministry of Health, Makkah, Saudi Arabia;; ^6^ Oxford University Clinical Research Unit, Faculty of Medicine Universitas Indonesia, Jakarta, Indonesia;; ^7^ Department of Medical Microbiology, Radboud University Medical Center, Radboud, the Netherlands;; ^8^ Instituto de Medicina Tropical Alexander von Humboldt, Universidad Peruana Cayetano Heredia, Lima, Peru;; ^9^ Tropical Medicine Foundation Dr Heitor Vieira Dourado, Manaus, Brazil;; ^10^ Department of Parasitology, Faculty of Medicine, University of Colombo, Colombo, Sri Lanka;; ^11^ Swiss Tropical and Public Health Institute, Allschwil, Switzerland;; ^12^ University of Basel, Basel, Switzerland;; ^13^ Bicol University College of Medicine, Legazpi City, Philippines;; ^14^ National Malaria Program, Ministry of Health, Phnom Penh, Cambodia;; ^15^ Independent Consultant, Bangkok, Thailand;; ^16^ Global Malaria Programme, WHO, Geneva, Switzerland

## Abstract

The WHO recommends that all affected countries work toward the elimination of malaria, even those still experiencing a high burden of disease. However, malaria programs in the final phase of elimination or those working to prevent re-establishment of transmission after elimination could benefit from specific evidence-based recommendations for these settings as part of comprehensive and quality-controlled malaria guidelines. The WHO convened an external guideline development group to formulate recommendations for interventions to reduce or prevent malaria transmission in areas with very low– to low-transmission levels and those that have eliminated malaria. In addition, several interventions that could be deployed in higher burden areas to accelerate elimination, such as mass drug administration, were reviewed. Systematic reviews were conducted that synthesized and evaluated evidence for the benefits and harms of public health interventions and summarized critical contextual factors from a health systems perspective. A total of 12 recommendations were developed, with five related to mass interventions that could be deployed at higher transmission levels and seven that would be most appropriate for programs in areas close to elimination or those working to prevent re-establishment of transmission. Four chemoprevention, two active case detection, and one vector control interventions were positively recommended, whereas two chemoprevention and three active case detection interventions were not recommended by the WHO. None of the recommendations were classified as strong given the limited and low-quality evidence base. Approaches to conducting higher quality research in very low– to low-transmission settings to improve the strength of WHO recommendations are discussed.

## INTRODUCTION

From 2000 to 2021, the number of countries reporting fewer than 10,000 malaria cases per year, a threshold that is nominally considered an indication of nearing elimination, increased from 26 to 46.^1^ During that same period, 23 countries eliminated malaria within their borders and 12 were certified malaria-free by the WHO. The *Global Technical Strategy for Malaria 2016–2030* includes the goal of eliminating malaria in an additional 35 countries by 2030^2^; in 2020, this milestone was reached by 10 countries despite a global slowdown in progress toward achieving goals for reductions in malaria morbidity and mortality rates.^3^

Recent achievements in malaria elimination were facilitated by the 2017 publication *A Framework for Malaria Elimination* (Framework), which provides guidance on the tools, activities, and dynamic strategies required to interrupt transmission and prevent re-establishment of malaria.^4^ The Framework emphasizes that every country, including those with a high burden of malaria, should consider malaria elimination as a goal and adjust interventions as needed and appropriate to accelerate progress. In that respect, the artificial divisions between control, pre-elimination, and elimination of malaria were erased. Malaria elimination is now viewed as a continuous process of tailoring and adjusting intervention mixes to optimize reductions in malaria morbidity and mortality in a given setting and monitoring changes in the epidemiology and transmission characteristics of malaria until transmission has declined to the point that intensive and focused elimination interventions can be implemented.

When malaria elimination is viewed as a continuous process that may start even when countries retain a substantial burden of disease, many of the interventions recommended to reduce the burden of disease and death due to malaria, such as case management, vector control, and chemoprevention, will also contribute to elimination through reductions in transmission. However, the epidemiology of malaria in the final stages of elimination and the potential for transmission during the post-elimination period when programs are working to prevent re-establishment necessitate interventions that may not be feasible, appropriate, or cost-effective when there is a higher burden of disease.

In the past, WHO recommendations for health policies were developed almost exclusively from expert consensus. However, as the global trend shifted to the use of standardized, evidence-based processes for guideline development, the WHO adopted internationally recognized methods and standards to ensure the quality of its guidelines.^5^ The key principles of the WHO guideline development process include clarity and transparency; multidisciplinarity; stakeholder input; minimization of the risk of bias; use of publicly available evidence; and systematic and comprehensive assessment of the balance of an intervention’s potential benefits and harms with explicit consideration for other relevant factors. A WHO guideline, such as the *WHO Guidelines for Malaria*, includes specific recommendations for clinical practice, public health programs, or health policies.^6^ Recommendations help the user of the guideline to decide whether to implement certain interventions and where and when to do so. However, the *WHO Guidelines for Malaria* clearly state that recommendations are not meant to be overly prescriptive and urge each malaria program to use local data as part of a problem-solving approach to determine whether recommendations are relevant to its local setting.

The systematic reviews presented in this 11-article supplement to the *Journal *were commissioned by the WHO and used in the formulation of the first evidence-based recommendations for interventions in the final phase of elimination and prevention of re-establishment of malaria.^6^ In addition, certain mass strategies (i.e., mass drug administration, mass relapse prevention, and mass testing and treatment) were included, as they are often implemented to drive transmission down over a short period of time to accelerate malaria elimination, even though they may be deployed at higher transmission settings before the final phase of elimination. The articles in this *Supplement* summarize the available evidence for the effect of key interventions on proxy measures of malaria transmission (incidence and prevalence in the general population) and for critical contextual factors that may modify the benefits and harms of the interventions or affect implementation and impact. The second paper in this *Supplement* describes the methods used in conducting the systematic reviews and meta-analyses and how the Grading of Recommendations, Assessment, Development and Evaluations (GRADE) approach was used to rate the certainty of evidence.^7^^,^^8^ This paper provides an overview of the WHO guideline development process, including the topics considered for development of recommendations, and discusses the experience of the authors, who were either members of the Guideline Development Group (GDG) convened by the WHO to develop recommendations for malaria elimination or members of the WHO Secretariat who supported the guideline development effort. The current official WHO recommendations on malaria can be found online in the *WHO Guidelines for Malaria*.^6^

## MATERIALS AND METHODS

A planning proposal for the development of malaria elimination recommendations was approved by the WHO Guideline Review Committee, which includes WHO staff appointed from across the organization to oversee and provide quality control for the guideline development process.^5^ An internal steering group, composed of WHO staff from the Global Malaria Programme, other relevant departments, and WHO regional offices, determined the scope of the elimination recommendations and drafted the key questions. The steering group selected the members of the GDG for their technical expertise related to research on malaria interventions or program implementation and balanced representation across WHO regions, areas of expertise, and gender.^6^ The steering group ensured appropriate management of potential conflicts of interests, which were publicly declared according to the organization’s policy. The 12 members of the GDG finalized the key questions, prioritized outcomes, and formulated recommendations based on a review of the evidence presented in the systematic reviews. A trained guideline development methodologist (E. A.) with experience in the WHO guideline development process worked with both the review teams and the GDG to support the GRADE of the evidence and the formulation of evidence-based recommendations by the GDG using an evidence-to-decision framework.^8^^,^^9^ Finally, an external review group provided feedback on the relevance of the key questions and conducted a final check of recommendations to identify any errors or missing data and to comment on the clarity of the wording.

### Scope of the malaria elimination recommendations.

The steering group identified 10 key questions regarding potential interventions relevant for malaria elimination, particularly in the final phase of elimination or prevention of re-establishment of transmission (Table 1). Key questions were developed from public health needs identified by national malaria programs and other stakeholders and were not driven by the anticipated availability of evidence. Each question was formulated in the population, intervention, comparison, and outcome (PICO) format to guide the systematic reviews and synthesis of evidence (Supplemental Table 1). The WHO Malaria Policy and Advisory Group reviewed and endorsed the scope of the elimination-related questions.^10^

**Table 1:** Key questions related to potential interventions for malaria elimination

Common Name	Key Question
Mass drug administration for *Plasmodium falciparum*	1A. Should people living in a defined geographical area be given a full therapeutic course of an antimalarial medicine at approximately the same time to reduce transmission of *P. falciparum*?
Mass drug administration for *Plasmodium vivax*	1B. Should people living in a defined geographical area be given a full therapeutic course of an antimalarial medicine at approximately the same time to reduce transmission of *P. vivax*?
Mass relapse prevention	2. Should people living in a defined geographical area be given a hypnozoiticide at approximately the same time to reduce transmission of *P. vivax*?
Mass testing and treatment	3. Should people living in a defined geographical area be tested for malaria at approximately the same time and treated if positive to reduce human malaria transmission?
Targeted testing and treatment	4. Should people at increased risk of malaria infection be tested for malaria and treated if positive to reduce human malaria transmission?
Targeted drug administration	5. Should people at increased risk of malaria infection be given a full therapeutic course of an antimalarial medicine to reduce human malaria transmission?
Testing and treatment at points of entry (border screening)	6. Should people crossing a point of entry be tested for malaria and treated if positive to reduce importation of human malaria parasites?
Reactive case detection and treatment	7. Should people residing with or near a confirmed malaria case be tested for malaria at approximately the same time and treated if positive to reduce human malaria transmission?
Reactive drug administration	8. Should people residing with or near a confirmed malaria case be given a full therapeutic course of an antimalarial medicine at approximately the same time to reduce human malaria transmission?
Reactive indoor residual spraying	9. Should the houses of people residing with or near a confirmed malaria case be sprayed with a residual insecticide to reduce human malaria transmission?

In settings where transmission occurs broadly across a landscape, albeit with variation in intensity, “mass” interventions that target the entire population may be needed to deplete the human parasite reservoir and reduce transmission. These interventions include mass drug administration, mass relapse prevention, and mass testing and treatment, and are represented by key questions 1 to 3 (Table 1).

As transmission declines, geographical clustering generally becomes more pronounced, and cases may become focalized among people with a higher level of exposure to infected mosquitoes than the general population.^11^^,^^12^ In settings where the majority of infections are accrued by a subset of the population with identifiable risk factors, interventions targeted to these groups may be a more acceptable and cost-effective approach to reducing transmission than targeting an entire geographical area.^13^ Identifiable risk factors could be demographic characteristics or occupations associated with outdoor or nighttime activities such as mining, playing sports, socializing or sleeping outdoors, engaging in forest activities such as rubber tapping, tending cattle, and serving in the military or police. “Targeted” strategies include targeted testing and treatment, targeted drug administration, and testing and treatment at points of entry (also known as border screening) and are reflected in key questions 4 to 6 (Table 1).

In the final phase of elimination, case-based surveillance and investigations at the residence of cases become feasible. Given the clustered nature of malaria cases at very low–transmission levels, interventions may be deployed around parasitologically confirmed malaria cases in an effort to further reduce or interrupt transmission. Referred to as “reactive” strategies because they are initiated in response to confirmed cases, these actions may target both household members and neighbors of an index case and all other individuals exposed to malaria transmission in the same manner as the index case, often through co-travel. The most common reactive strategies are reactive case detection and treatment, reactive drug administration, and reactive indoor residual spraying and are the subject of key questions 7 to 9 (Table 1).

Several of the interventions (mass drug administration, mass relapse prevention, targeted drug administration, and reactive drug administration; questions 1A, 1B, 2, 5, and 8; Table 1) included in the scope of the malaria elimination recommendations are chemoprevention strategies, defined as the provision of full therapeutic courses of antimalarial medicines at prescheduled times (or at the time an index case is identified), irrespective of infection status, to treat existing infections and prevent new infections over the prophylactic period of the antimalarial medicine. The key questions also included active case detection and treatment strategies (mass testing and treatment, targeted testing and treatment, testing and treatment at points of entry, and reactive case detection and treatment; key questions 3, 4, 6, and 7; Table 1). One of the interventions (reactive indoor residual spraying) is a vector control intervention (key question 9; Table 1).

### Population, intervention, comparison, and outcome question formulations.

Population, intervention, comparison, and outcome questions to guide the systematic review were deliberately formulated such that the GDG’s answer to the questions (on the basis of the systematic review, consideration of contextual factors, and GDG members’ judgments) would form the recommendation (Table 1). The population for all PICO questions included both adults and children. All questions were relevant to areas with ongoing transmission or where the risk of transmission would remain after elimination (i.e., areas with potential transmission; Supplemental Table 1), except for the questions related to mass drug administration, which were limited to areas of ongoing transmission. The comparator in all cases was “no intervention,” which meant the absence of the intervention under consideration rather than the absence of all malaria interventions: It was expected that most studies would be conducted against a background of interventions that were the standard of care in the area.

To capture the beneficial effect of interventions on transmission and progress toward elimination, primary outcomes (malaria incidence and prevalence) were measured at the population level rather than only among those directly participating in the intervention. Community-level outcomes included the incidence or prevalence of infection or disease due to human malaria species, but some questions were specific for *Plasmodium* *falciparum* or *Plasmodium vivax*. The clarification that only “human” malaria species were to be considered was meant to signal that recommendations would not extend to situations where there was spillover transmission from nonhuman primate malaria species; separate recommendations are needed for these situations. Potential harms resulting from the intervention were generally measured among those who received the intervention, such as side effects from the medicine administered, although some were measured at the community level, such as drug or insecticide resistance. Before the systematic reviews were presented, the GDG ranked the outcomes for each question to identify the priority outcomes. In addition, the GDG identified and ranked additional factors that potentially could alter either the beneficial or harmful impacts of the intervention (i.e., potential effect modifiers). If there were sufficient studies, the systematic review teams were asked to quantitatively evaluate the top five potential effect modifiers per question.

### Contextual factors.

The WHO considers several additional factors beyond benefits and harms of an intervention to determine the direction and strength of a recommendation.^5^ These additional factors are referred to as “contextual,” as they provide a health systems perspective for the development of recommendations.^4^ The following main contextual factors are considered by the WHO:

1. Values and preferences of the affected population: The relative importance assigned to outcomes by those affected should inform the prioritization of outcomes.
2. Resource implications: The resource requirements of an intervention, particularly in relation to the extent of benefits, influence the strength and direction of a recommendation. However, recognizing that costs, resource needs, and impact may vary over time and space, the absolute values were considered less important than the qualitative assessment of the likely affordability of the intervention.
3. Equity and human rights: Interventions that result in substantial health inequities or harm human rights are not likely to be recommended, whereas those that improve health equity are more likely to receive a positive recommendation.
4. Acceptability: Interventions that are more acceptable to all or most stakeholders are more likely to be recommended.
5. Feasibility: The ability to implement the intervention in a real-world setting will affect the strength and direction of the recommendation.

The review teams were asked to identify articles contributing evidence to any of these areas among the articles identified in their systematic review. However, no specific systematic literature searches and no assessments of the quality of evidence were undertaken for contextual factors.

### Systematic reviews.

The WHO commissioned the Barcelona Institute for Global Health (Barcelona, Spain) and the U.S. Centers for Disease Control and Prevention (Atlanta, GA) to conduct the systematic reviews and data syntheses and meta-analyses that are included in this *Supplement*. The review teams developed protocols based on best practices for systematic reviews, consulted with the steering group and guideline development methodologist to clarify aspects of the key questions and methodology, and shared protocols within and across the two institutions to harmonize approaches, particularly with respect to inclusion and exclusion criteria for studies.

The methods for the systematic reviews are reported in the second paper in this *Supplement*, with any deviations noted in the methods of the specific reviews.^7^ In brief, electronic databases were searched for relevant studies based on prespecified search terms developed in collaboration with librarians at each agency. Although the two review teams used different approaches to assess the risk of bias in individual studies, both used the GRADE approach to determine the level of certainty of the evidence, whereas the guideline development methodologist and steering group helped ensure a consistent approach to the presentation of evidence.^8^ The PRISMA (Preferred Reporting Items for Systematic Reviews and Meta-Analyses) checklist was used for all systematic reviews.^14^

### Formulation of recommendations.

The GDG completed the GRADE evidence-to-decision framework for each recommendation to provide clear, concise, and transparent explanations of the evidence reviewed and the judgments made by the GDG with respect to the balance of benefits and harms and consideration of contextual factors.^9^ Evidence from the systematic reviews was complemented by the expertise and experience of GDG members, particularly for areas in which the evidence base was limited or nonexistent. The GDG formulated recommendations through consensus. The strength and direction of recommendations were linked to the certainty of evidence for benefits and harms but could be modified by the GDG’s judgments related to contextual factors. The direction of the recommendation was either for or against the intervention, and the strength of a recommendation (i.e., conditional or strong) reflected the GDG’s level of confidence that the desirable effects of an intervention outweighed the negative effects. The interpretations of the strength and direction of recommendations from the perspectives of policymakers, program managers, and end users are provided in Table 2.

**Table 2:** Interpretation of WHO malaria recommendations according to strength and direction^6^

Strength and Direction of Recommendation	Interpretation	
	For Policymakers and Program Managers	For End Users
Strongly for	This recommendation can be adopted as policy in most situations.	Most people in this situation would want the recommended intervention, and only a small proportion would not.
Conditionally for	The recommended intervention can be adopted as a policy after relevant stakeholders judge its positive consequences as outweighing its negative ones based on a careful assessment of the contextual factors.	The majority of people in this situation would want the recommended intervention, but many would not.
Conditionally against	The recommended intervention should not be adopted as a policy unless relevant stakeholders judge its positive consequences as outweighing its negative ones based on a careful assessment of the contextual factors.	The majority of people in this situation would not want the intervention, but many would.
Strongly against	This recommendation should not be adopted as policy in most situations.	Most people in this situation would not want the intervention, and only a small proportion would.

For each recommendation, the GDG provided comments that described additional considerations for when and where interventions might be most appropriate. Consistent with the principle that interventions should be tailored to the local setting, the GDG was careful to phrase recommendations so they would apply to any size area and not necessarily to an entire country. The GDG also developed a list of topics for further research when it was felt that more evidence could help strengthen or clarify the recommendations. The final guideline document was drafted by the steering group, reviewed for clarity by the external review group, finalized by the GDG, and published by the WHO.^6^ The WHO uses an online platform, MAGIC authoring and publication platform (MAGICapp), to facilitate frequent updates and to ensure that the most recent version of recommendations is clear and available. The guidelines can be printed in pdf format or accessed online or through the Malaria toolkit app, an application available on mobile devices.

## FINDINGS AND RECOMMENDATIONS FOR POLICY AND PROGRAM

Upon completion of the initial data synthesis, significant heterogeneity was identified among studies reporting on mass drug administration for *P. falciparum* (key question 1A; Table 1) that was found to be reduced through stratification of studies by level of transmission intensity. As a result, question 1A was further divided into areas of very low to low and moderate to high transmission (Supplemental Table 1).

After reviewing evidence from the systematic reviews, the GDG modified the PICOs for several questions. The GDG felt that the setting for strategies targeted to persons at increased risk of malaria infection (i.e., targeted drug administration and targeted testing and treatment; key questions 2, 4, and 5; Table 1) should be limited to areas of very low to low transmission or post-elimination and modified the PICO questions accordingly (Supplemental Table 1). In addition, the GDG determined that the question on testing and treatment at points of entry (i.e., border screening; key question 6; Table 1) should be divided into two questions: routine testing and treatment of people entering an area nearing elimination and testing and treatment of organized or identifiable groups shortly after arriving or returning from malaria-endemic areas. As a result, recommendations were developed separately for each intervention.

The research evidence reviewed by the GDG is presented in the *WHO Guidelines for Malaria* along with detailed judgments made by the GDG regarding each element of the evidence-to-decision framework, an overall justification, and comments related to the implementation of the intervention.^6^ A summary of the recommendations is presented in Table 3. The recommendations favored chemoprevention strategies over active case detection interventions; although active case detection interventions were generally considered more acceptable and feasible to implement, the available evidence suggested that they were not as effective at reducing transmission as chemoprevention interventions. However, the GDG recognized that active case detection strategies may serve a purpose other than to directly reduce transmission of malaria. For example, active case detection could contribute to improving the sensitivity or timeliness of surveillance systems or to extending surveillance and case management to underserved populations. The GDG’s recommendations on active case detection interventions do not cover situations in which objectives are related to improving surveillance; reference in these situations should be made to the WHO surveillance guidance.^15^

**Table 3:** Summary of WHO recommendations for malaria transmission reduction and the final phase of elimination and prevention of re-establishment*

Approach	Type of Intervention	WHO suggests/recommends:			WHO does not suggest/recommend:		
		Intervention	Certainty of Evidence	Strength of Recommendation	Intervention	Certainty of Evidence	Strength of Recommendation
Mass	Chemoprevention	Mass drug administration to reduce transmission of *Plasmodium falciparum* in very low– to low- transmission settings	Low	Conditional	Mass drug administration to reduce transmission of *P. falciparum* in moderate- to high-transmission settings†	Very low	Conditional
Mass	Chemoprevention	Mass drug administration to reduce transmission of *Plasmodium vivax*	Very low	Conditional	Mass treatment with an 8-aminoquinoline medicine alone to reduce transmission of *P. vivax*	Very low	Conditional
Mass	Active case detection	–	–	–	Mass testing and treatment to reduce the transmission of malaria	Moderate	Conditional
Targeted	Chemoprevention	Targeted chemoprevention to people with increased risk of infection in areas with very low– to low-transmission or post-elimination settings	Very low	Conditional	–	–	–
Targeted	Active case detection	–	–	–	Targeted testing and treatment of people with increased risk of infection	Very low	Conditional
Targeted	Active case detection	Testing and treatment of organized or identifiable groups arriving or returning from malaria-endemic areas to reduce importation of malaria in areas approaching elimination or post-elimination settings	Very low	Conditional	Routine malaria testing and treatment of people arriving at points of entry (land, sea, or air) to reduce importation	Very low	Conditional
Reactive	Active case detection	Testing and treatment of people living with or near a confirmed malaria case in areas approaching elimination or post-elimination settings	Very low	Conditional	–	–	–
Reactive	Chemoprevention	Chemoprevention to people residing with or near a confirmed malaria case in areas approaching elimination or post-elimination settings	Very low	Conditional	–	–	–
Reactive	Vector control	Indoor residual spraying of houses of a confirmed malaria case and neighbors in areas approaching elimination or post-elimination settings	Very low	Conditional	–	–	–

GDG = Guideline Development Group.

* The full evidence-to-decision framework, including justifications for the judgments of the GDG, and detailed evidence reviews can be found in the *WHO Guidelines for Malaria*.^6^ The *WHO Guidelines for Malaria* clearly state that recommendations are not meant to be overly prescriptive and urge each malaria program to use local data in a problem-solving approach to determine whether recommendations are relevant to its local setting.

† The WHO has made a conditional recommendation in favor of mass drug administration to reduce disease burden in moderate- and high-transmission settings.

Historically, mass drug administration has been used as an intervention either to reduce malaria disease burden or to reduce malaria transmission, but the distinction between these two use cases for *P. falciparum* is to some extent artificial. Any intervention that reduces transmission will also reduce disease burden, and burden-reducing interventions that reach a sufficient proportion of the population will also reduce transmission. The question of mass drug administration was considered not only by the GDG on elimination but also by a separate GDG that reviewed chemoprevention strategies more generally. Both GDGs broadly recommended that programs may consider mass drug administration to reduce *P. falciparum* transmission in very low– to low-transmission settings, and the GDG on chemoprevention recommended mass drug administration additionally to reduce disease burden in moderate- to high-transmission settings.^6^ Malaria programs should therefore review the mass drug administration recommendations and practical information for both burden and transmission reduction settings and decide whether a mass drug administration intervention is likely to lead to a successful outcome in their context.

When recommendations might be strengthened or changed based on new evidence, the GDG identified research areas for further investigation, and these can be found in the online version of the guidelines under the “More information” tabs^6^; investigators seeking to generate policy-relevant research are encouraged to review these questions.

## LIMITATIONS

The evidence base for the malaria elimination interventions included in these guidelines was limited by either a paucity of high-quality studies or a complete lack of eligible studies, as in the case of targeted testing and treatment. The generally low number of studies precluded the possibility of conducting subgroup analyses to analyze the effect of potential effect modifiers, except in the case of mass drug administration for *P. falciparum*, where transmission level could be used to stratify the results. The GDG was transparent about decisions made based on its expertise and experience rather than on empirical evidence and frequently stressed the need for the malaria community to deploy more robust study designs, although they recognized the challenges to conducting robust impact evaluations in very low– or low-transmission settings. The final result was that none of the recommendations for elimination or prevention of re-establishment was considered to be strong.

In addition to the lack of empirical evidence for epidemiological impact of interventions, evidence for contextual factors was also limited. In particular, few outcomes were disaggregated by gender or socioeconomic status, which prevented an evidence-based determination of intervention impact on health equity. Evidence for acceptability and feasibility was more common, and although cost data could be found in some studies, cost-effectiveness data were rare, resulting in part from the difficulty of measuring intervention effectiveness in areas approaching elimination.

## IMPROVING THE QUALITY OF EVIDENCE FOR MALARIA ELIMINATION INTERVENTIONS

It is challenging to implement high-quality studies of interventions in very low– to low-transmission settings, as relatively large sample sizes are required to detect an effect when the frequency of the outcome is very low. Generalizing results of interventions from higher to lower transmission settings may not be valid owing to differences in population levels of immunity and spatiotemporal transmission patterns, among other factors that may vary between areas approaching elimination and those with higher levels of transmission. To generate higher quality evidence despite these challenges, the malaria research community could improve the design and implementation of nonrandomized studies in very low– to low-transmission settings. The Cochrane collaborative has developed the ROBINS-I (Risk of Bias in Nonrandomized Studies – of Interventions) tool to assess bias in quasi-experimental studies; this tool could be used to guide the planning and reporting of nonrandomized studies to improve the certainty of evidence.^16^ In particular, three potential sources of bias that are eliminated through randomization are of particular concern for quasi-experimental or observational studies (i.e., confounding, selection bias, and misclassification of intervention status) and should be considered carefully in the design phase. The certainty of evidence can also be improved by ensuring that published reports provide all the data needed to evaluate these and other potential sources of bias. In addition, malaria researchers are encouraged to use the *WHO Malaria Guidelines* to aid in the design of future research studies to improve the evidence base for global policies.^6^ This includes consideration of the PICO questions that the WHO utilizes to define the scope of systematic reviews and the inclusion and exclusion criteria that determine which research studies are included in the data synthesis.

In very low– to low-transmission settings where malaria surveillance may be considered reasonably sensitive (i.e., a malaria case is likely to be detected, should one occur), intervention studies could take advantage of routine malaria surveillance systems to reduce the cost of collecting outcome data. Interrupted time series are an underutilized, quasi-experimental study design that could be used to evaluate the impact of programmatic interventions, particularly those expected to have a community impact, that start at a defined point in time.^17^^,^^18^ Given the wealth of experience they have gained and the new approaches they are testing, national malaria elimination programs should receive support to formulate research questions and to design, implement, analyze, and publish robust evaluations of their elimination interventions so they can become part of the evidence base informing WHO recommendations.

## CONCLUSION

As more countries seek to eliminate malaria, the need for evidence-based recommendations grows more urgent. Based on a relatively scarce body of evidence, the WHO has developed the first set of recommendations for areas approaching elimination and those in the post-elimination phase. These new recommendations are being presented through online platforms, smartphone apps, and an online training curriculum to improve dissemination and uptake.^19^ The WHO is tracking uptake and engaging with stakeholders to continually improve the development of recommendations in its malaria guidelines. For their part, malaria researchers and national elimination programs are encouraged to consider the criteria for evidence that may be included in WHO recommendations and suggestions for stronger study designs as they plan research or program evaluations. By improving the quality of the evidence base, existing recommendations can be strengthened, and new recommendations can be generated.

## Supplemental Materials

10.4269/ajtmh.22-0768 Supplemental Materials
