# Development of WHO Recommendations for the Final Phase of Elimination and Prevention of Re-Establishment of Malaria

**DOI:** 10.4269/ajtmh.22-0768cor

**Published:** 2025-01-08

In the original research paper, “Development of WHO Recommendations for the Final Phase of Elimination and Prevention of Re-Establishment of Malaria” by Marsh and others (https://doi.org/10.4269/ajtmh.22-0768), the authors missed to correct the data of the Certainty of Evidence (Column 4), prior to publishing. Two of the interventions listed (Reactive – Chemoprevention and Reactive – Vector Control) were both mislabeled as “very low” but the former should have been labeled as “low” and the latter as “moderate” per the WHO guidelines (WHO Guidelines for Malaria - October 16, 2023).

The paper has therefore been corrected. The results and conclusions are unchanged. A summary of the corrected data is shown below:

Reported Certainty of Evidence Levels for Reactive Approach of Chemoprevention listed as Very Low was changed to Low.
Reported Certainty of Evidence Levels for Reactive Approach of Vector Control listed as Very Low was changed to Moderate.
(TABLE 3, page 8)

