# Supplementary material for: Development of WHO Recommendations for the Final Phase of Elimination and Prevention of Re-Establishment of Malaria
Source: Am J Trop Med Hyg. 2023 Dec 20;110(4 Suppl):3–10. doi: 10.4269/ajtmh.22-0768 (PMC10993787; doi:10.4269/ajtmh.22-0768)
Supplement: Supplemental Materials [file tpmd220768.SD1.pdf]

Supplemental Table 1. Initial list of key questions for the development of elimination recommendations by WHO defined by population, intervention, comparison and outcome.

Population	Intervention	Comparison	Outcome
1. A. Should people living in a defined geographical area be given a full therapeutic course of an antimalarial medicine at approximately the same time to reduce transmission of *P. falciparum*?*			
Adults and children residing in an area with ongoing *P. falciparum* transmission	Administration of a full therapeutic course of an antimalarial medicine to the entire population of a delimited geographical area at approximately the same time	No intervention	Measured at the community level:<br>Incidence of malaria infection<br>Prevalence of malaria infection<br>Incidence of clinical malaria<br>Prevalence of drug resistance markers<br>Measured among those who participated in the study:<br>Adverse events<br>Prevalence of drug resistance markers
1. B. Should people living in a defined geographical area be given a full therapeutic course of an antimalarial medicine at approximately the same time to reduce transmission of *P. vivax*?			
Adults and children residing in an area with ongoing *P. vivax* transmission	Administration of a full therapeutic course of an antimalarial medicine (schizonticide) with or without a hypnozoiticide to the entire population of a delimited geographical area at approximately the same time	No intervention	Measured at the community level:<br>Incidence of malaria infection<br>Prevalence of malaria infection<br>Incidence of clinical malaria<br>Prevalence of drug resistance markers<br>Measured among those who participated in the study:<br>Adverse events<br>Prevalence of drug resistance markers
2. Should people living in a defined geographical area be given a hypnozoiticide at approximately the same time to reduce transmission of *P. vivax*?			
Adults and children residing in an area with ongoing or potential *P. vivax* transmission	Administration of a full therapeutic course of an antimalarial medicine (hypnozoiticide) to the entire population of a delimited	No intervention	Measured at the community level:<br>Incidence of malaria infection<br>Prevalence of malaria infection<br>Incidence of clinical malaria

	geographical area at approximately the same time		Measured among those who participated in the study:<br>Adverse events<br>Prevalence of drug resistance markers
3. Should people living in a defined geographical area be tested for malaria at approximately the same time and treated if positive to reduce human malaria transmission?			
Adults and children residing in an area with ongoing or potential human malaria transmission	Parasitologic testing of the entire population of a delimited geographical area at approximately the same time and treatment of confirmed malaria cases	No intervention	Measured at the community level:<br>Incidence of malaria infection<br>Prevalence of malaria infection<br>Incidence of clinical malaria<br>Prevalence of drug resistance markers<br>Measured among those who participated in the study:<br>Adverse events<br>Prevalence of infection<br>Prevalence of drug resistance markers
4. Should people at increased risk of malaria infection be tested for malaria and treated if positive to reduce human malaria transmission?†			
Adults and children at increased risk of malaria infection residing in areas with ongoing or potential human malaria transmission	Parasitologic testing of everyone at risk and treatment of confirmed malaria cases	No intervention	Measured at the community level:<br>Incidence of malaria infection<br>Prevalence of malaria infection<br>Incidence of clinical malaria<br>Prevalence of drug resistance markers<br>Measured among those who participated in the study:<br>Adverse events<br>Prevalence of infection
5. Should people at increased risk of malaria infection be given a full therapeutic course of an antimalarial medicine to reduce human malaria transmission?†			

Adults and children at increased risk of malaria infection residing in areas with ongoing or potential human malaria transmission	Administration of a full therapeutic course of an antimalarial medicine (schizonticide) with or without a hypnozoiticide to everyone at risk	No intervention	Measured at the community level:<br>Incidence of malaria infection<br>Prevalence of malaria infection<br>Incidence of clinical malaria<br>Prevalence of drug resistance markers<br>Measured among those who participated in the study:<br>Adverse events
6. Should people crossing a point of entry be tested for malaria and treated if positive to reduce importation of human malaria parasites?‡			
Adults and children entering or returning to a country or subnational area with ongoing or potential human malaria transmission	Parasitologic testing of everyone crossing a point of entry and treatment of confirmed malaria cases	No intervention	Measured at the community level:<br>Number of positive cases as a proportion of total imported cases<br>Measured among those who participated in the study:<br>Adverse events<br>Prevalence of infection
7. Should people residing with or near a confirmed malaria case be tested for malaria at approximately the same time and treated if positive to reduce human malaria transmission?			
Adults and children residing in an area with ongoing or potential human malaria transmission	Parasitologic testing of people living with or near a confirmed malaria case at approximately the same time and treatment of confirmed malaria cases	No intervention	Measured at the community level:<br>Incidence of malaria infection<br>Prevalence of malaria infection<br>Incidence of clinical malaria<br>Measured among those who participated in the study:<br>Adverse events<br>Prevalence of infection
8. Should people residing with or near a confirmed malaria case be given a full therapeutic course of an antimalarial medicine at approximately the same time to reduce human malaria transmission?			
Adults and children residing in an area with ongoing or potential human malaria transmission	Administration of a full therapeutic course of an antimalarial medicine (schizonticide) with or without a hypnozoiticide to people living	No intervention	Measured at the community level:<br>Incidence of malaria infection<br>Prevalence of malaria infection<br>Incidence of clinical malaria

	with or near a confirmed malaria case at approximately the same time		Measured among those who participated in the study:<br>Adverse events<br>Prevalence of infection
9. Should the houses of people residing with or near a confirmed malaria case be sprayed with a residual insecticide to reduce human malaria transmission?			
Adults and children residing in an area with ongoing or potential human malaria transmission	Spraying a residual insecticide on the inside surfaces and ceilings of the houses of a confirmed malaria case and neighbors	No intervention	Measured at the community level:<br>Incidence of malaria infection<br>Prevalence of malaria infection<br>Incidence of clinical malaria<br>Insecticide resistance<br>Measured among those who participated in the study:<br>Adverse events

*Question was split into settings of very low to low and moderate to high transmission after significant heterogeneity in the meta-analysis was found to be reduced by stratification by level of transmission.

†The GDG decided that strategies targeted to persons at increased risk of malaria (i.e. targeted interventions) should be limited to areas of very low to low transmission and used a modified PICO question.

‡ The GDG added testing and treatment of organized or identifiable groups shortly after arriving or returning from malaria-endemic areas as an intervention under border screening.
